# Library services enriching community engagement for dementia care: The Tales & Travels Program at a Canadian Public Library as a case study

**DOI:** 10.1177/09610006211065170

**Published:** 2021-12-29

**Authors:** Jiamin Dai, Joan C. Bartlett, Karyn Moffatt

**Affiliations:** McGill University, Canada

**Keywords:** Community-based research, community information services, dementia care, librarianship research and practice, public libraries

## Abstract

Growing dementia-friendly library services are contributing to community-based dementia care. Emerging community programs in libraries and museums provide notable opportunities for promoting engagement and inclusivity, but these programs have yet to receive in-depth assessments and analyses to guide future research and practice. This paper presents a case study examining a social and storytelling program for people with dementia run by a Canadian public library. It investigates two research questions: How can public library programs contribute to community-based dementia care? And what are public libraries’ strengths and challenges in running programs for people with dementia? The study involves participant observations of the program and semi-structured interviews with people with dementia, caregivers, and program facilitators (librarians and Alzheimer Society coordinators). Through thematic analysis of fieldnotes and transcripts, the study reveals how this inclusive platform supports engagement, fosters relationships, helps caregivers, and reaches broader communities. This research further uncovers the librarians’ diversified roles as demonstrated through their collaboration with professionals, preparation and research, and facilitation of the sessions. This paper advances librarianship research on enriching community-based dementia care, including furthering inclusivity and engagement and extending accessible library services. By analyzing library programming for the dementia community and assessing its strengths and challenges, the paper highlights librarians’ awareness of the community’s evolving needs and their collaboration with other professionals. It offers practical insights on useful resources and emerging best practices that will hopefully inspire other initiatives in which information professionals can help improve the well-being of vulnerable populations.

## Introduction

How can libraries help with healthcare issues such as dementia? With dementia care consuming considerable monetary and caregiving resources, a policy preference is to enable people with dementia to live at home, instead of in facilities, for as long as possible (Alzheimer Society of Canada, 2016; Alzheimer’s Association, 2020). Non-pharmacological approaches to improve home-based interventions are integral to dementia care services (Callahan et al., 2013), while the inclusive engagement of persons with dementia and their caregivers has positively affected interventions (Parker et al., 2008). This context reveals the necessity of involving both care recipients and providers in community-based activities. As a cornerstone of community services, public libraries are uniquely positioned to aid people with dementia and their families in reducing social isolation and maintaining quality of life.

Librarianship practices have increasingly engaged in community and library services for dementia care. The International Federation of Library Associations and Institutions (IFLA) provided “Guidelines for Library Services to Persons with Dementia” over a decade ago (Mortensen and Nielsen, 2007). With growing awareness of dementia among information professionals and the general public, recent publications have discussed expanding library customer services for the dementia community (Dickey, 2020). Notably, the Tales and Travel Memories, a storytelling and social program initiated in the Gail Borden Public Library District in Illinois, has showcased how librarians can join the care team for people with dementia (Riedner, 2015) and work toward building dementia-friendly libraries (Riedner et al., 2018). It also offers toolkits for librarians and caregivers under Creative Commons licensing.

This case study is situated in an adaption of the original Tales and Travel Memories series run by the Westmount Public Library in greater Montreal, Canada, in collaboration with the Alzheimer Society of Montreal. This Tales & Travels program runs for several series of five weekly sessions throughout the year. It invites participants (usually with early to middle stage dementia) to learn about a country each session through multi-sensory experiences. This group setting encourages people with dementia to share stories and memories while socializing with each other, caregivers, and facilitators. Each session consists of a story time, a coffee break featuring themed snacks, and a video viewing. This paper analyzes the contributions of such library programming to the dementia community and highlights its strengths and challenges.

## Background

### Library services as community-based programs

Public libraries have increasingly been expanding partnerships with various organizations through community-based programs. For example, collaborative workshops involving library and preschool staff have successfully cultivated deeper partnerships in supporting early learning and family engagement (Campana et al., 2020). Library participation has been highlighted in a multi-agency initiative for third-grade reading proficiency through a collective impact model (Pasini, 2018). In addition to supporting literacy skills, public library storytimes can create an early math learning environment, adding to the richness and broadness of library programs serving the communities (Campana, 2020). These recent collaborations in librarianship research and practice have demonstrated libraries’ essential role as community partners, potentially reaching further than early education.

The contributions and potentials of libraries in community building have been advocated and envisioned through such lenses as diversity, comprehensiveness, and transformation, calling for forging strong partnerships in the community and early involvement in policymaking (de la Peña McCook, 2000). Opening library spaces to community activities and partnering with other public services help position libraries as a key resource in community engagement (Goulding, 2009). Librarians’ increased participation in community development can address the needs of diverse population groups in specific contexts, more meaningfully impacting community members’ lives (Mehra, 2005).

### Dementia-friendly library services

To better serve the dementia community, librarianship researchers and practitioners have started to tackle communication barriers and develop best practices for customers at different stages of dementia (Dickey, 2020). Examining professional communication guidelines from library reference work and dementia care, prior work has uncovered an ethic of care “that manifests attentiveness, responsibility, responsiveness, and competence” (Dalmer and Campbell, 2020: 8). Emerging in communities around the world, dementia-friendly library services are extending access and equity to people with dementia, enabling them to contribute and enjoy group activities as valued members (Diller, 2017).

Library services offer opportunities for bridging the social isolation gap faced by people with dementia and their caregivers, touching upon socializing aspects for dementia care. Dedicated book groups have encouraged reading and community inclusiveness, promoting the benefits of reading for empathy, belonging, and civic participation (Baker et al., 2018). Informal social programming, such as Memory Cafés, has built upon reminiscing and storytelling to foster hospitality and interpersonal connection (Riedner et al., 2020). Our earlier work, which focused on identifying technological opportunities for promoting community-based social sharing, found that Tales & Travels provides effective agencies for social interaction, as well as an open environment that sustains a sense of belonging and normality (Dai and Moffatt, 2020).

### Emerging community-based programs for dementia care

Recent years have seen a growing number of community-based programs for dementia care in libraries and museums. In addition to reading and storytelling activities, libraries are exploring space for sensory stimulation through the creative use of tactile objects such as an interactive balance beam, a musical vibration bench, and touch-sensitive walls (Damron, 2019). Some libraries are partnering with local quilting clubs to create “fidget quilts” with aprons, zippers, and buttons to reduce anxiety (Brewer, 2018). Museums have mobilized art collections and spaces to offer specialized guided tours and creative workshops, supporting social interaction for people with dementia and respite for their caregivers in an esthetically pleasing environment (Montreal Museum of Fine Arts, 2018). These programs resonate with prior work on activity programming for dementia day services that has stressed the integration of physical settings with personalized, therapeutic experiences (Moore et al., 2006), calling for further examinations to facilitate more positive outcomes (Kinsey et al., 2021).

General-purpose guides already exist for communicating with and caring for people with dementia, (e.g., Coste, 2003; Mace and Rabins, 2017), and information professionals have started to adapt these guidelines for the specific context of library services. Emerging programs have been documented in reports, short papers, and magazine articles as reviewed above, but in-depth assessments and analyses are lacking in research-oriented library literature. While previous evaluations have examined such programs with a focus on quantitative measures (Tales and Travel Memories, n.d.), this qualitative case study offers detailed accounts from librarians, Alzheimer Society professionals, people with dementia, and their primary family caregivers, as well as participant observations of Tales & Travels. The findings provide an in-depth understanding of dementia-related library services through the lens of promoting engagement and inclusivity, aiding in future research and programming situated in the broader landscape of collaborative partnerships in community building.

## Method

This paper presents a case study investigating two research questions (RQs):

RQ1: How can public library programs contribute to community-based dementia care?
RQ2: What are public libraries’ strengths and challenges in running programs for people with dementia?

### Study design

This case study consists of three parts.

(1) Participant observations of the Tales & Travels sessions, focusing on participants’ verbal and nonverbal cues while socializing and interacting with various materials, as well as how facilitators maintain conversations and mediate emerging challenges. Each two-hour session involved story time with books and images, a coffee break with snacks related to the theme country, and video time watching clips about the country. The observational approach enhanced the quality of the data collection and interpretation, as well as ensuring the research was grounded in the community social context (DeWalt and DeWalt, 2011).
(2) Semi-structured interviews with Tales & Travels facilitators, discussing their perspectives on running a social program for people with dementia, including what successes and setbacks they experienced and how they envisioned improving the program. The semi-structured approach allowed for a consistent set of data, while maintaining flexibility for a deeper understanding of interviewees’ traits and perspectives.
(3) Semi-structured joint dyadic interviews with people with early-middle stage dementia and their primary family caregivers (e.g. the spouse), and individual interviews with primary family caregivers (when the person with dementia was unavailable). These interviews explored the experience of attending social activities as or with a person with dementia, including changes to their social lives and the social events they encountered. In particular, the joint dyadic interviews allowed for hearing the voices of both care-recipients and caregivers in the same conversation, as well as valuing the interdependence within each dyad (Caldwell, 2014).

Prior to data collection, the first author was a registered volunteer for the Alzheimer Society and volunteered for nine Tales & Travels sessions from February 2018 to March 2019. This preliminary work aided in understanding the procedures, getting acquainted with the participants, and becoming involved in the community; this minimized intrusiveness during observations (Creswell and Poth, 2017). Prolonged community engagement enabled the tailoring of interview questions to different caregiving situations and professional backgrounds (Dai and Moffatt, 2021).

The University Research Ethics Board reviewed and approved this research. Care was taken to attend to the decision-making capacity and consent for the participants with dementia, including involving them to the greatest extent possible in the process and respecting their verbal or physical expression of assenting to, or dissenting from, participation in research. The established consent protocol of involving vulnerable populations was followed: If participants with dementia were competent to consent (which was the case for many people with early-stage dementia), they would sign their own consent form; if they were not competent to consent (e.g. they already had a Power of Attorney in effect), the consent would be sought and maintained from the authorized third party (i.e. their legal guardian) that was not a member of the research team nor in a position of conflict of interest. However, in our case, all participants were competent to consent and signed their own consent form.

### Data collection

The observations and interviews ran concurrently from March to July 2019. Participant recruitment for observations started with the first author discussing the details with the library director and the librarian in charge of the program. The librarian introduced the project to attendees, identified which tables were open to observation, and assigned the first author to a table (without identifying which tables, if any, declined participation). Recruitment for facilitator interviews occurred in person and via email. Recruitment for dyad and caregiver interviews was mainly through word of mouth, especially at Tales & Travels.

Across eight Tales & Travels sessions, 11 people with dementia were observed. As a participant observer, the first author did not actively engage in the conversations but politely responded when approached. This moderate participation enabled her to conduct structured observation with limited interference at the scene, as a peripheral member of the group to acquire first-hand insights (DeWalt and DeWalt, 2011). She used a pen-and-paper-based observation guide and took detailed field notes non-intrusively, without collecting any identifying information. After each session, the field notes were promptly expanded both descriptively and reflectively to approximately 13,000 words in total.

The facilitator interviews (F1–F4) involved two males and two females, aged 27–32. F1 and F4 were librarians (master’s degrees in library and information studies); F2 and F3 were Alzheimer Society coordinators (degree/diploma in psychology and special care counseling). At the time of the interview, the most experienced facilitator had run 43 sessions, and others had separately facilitated nine, 15, and 24 sessions.^1^ We interviewed all the regular facilitators available, observing and interacting with all four during preliminary work and data collection. These interviews focused on the facilitators’ procedural work before, during, and after Tales & Travels sessions, as well as their reflections on dementia-related community social events through the lens of their professional expertise.

Five dyadic interviews and three individual caregiver interviews (C4, C5, and C8) were conducted (Table 1). C4’s spouse was present but did not participate. All the dyads interviewed (including C4 and her spouse) were living together at home; C5 and C8 were primary caregivers of parents with dementia living at facilities. All respondents shared their various social experiences to paint a comprehensive picture of their social lives and community-based activities including but not limited to Tales & Travels. Dyadic interviews focused on the pairs’ preferences and difficulties in social events, and individual caregiver interviews reflected caregiving perspectives about keeping people with dementia socially active.

**Table 1.** Dyad and caregiver background.

ID	Gender (age)	Relationship	Dementia condition	Experience with Tales & Travels
P1	M (84)	Spouses	Mid-stage Alzheimer’s	Attended regularly
C1	F (74)			
P2	M (90)	Spouses	Mid-stage Alzheimer’s	Attended once
C2	F (78)			
P3	F (80)	Neighbors	Mid-stage vascular	Attended regularly
C3	F (52)			Attended occasionally
P6	M (76)	Spouses	Mid-stage frontal temporal	None
C6	F (70)			
P7	F (81)	Common-law companions	Early/mid-stage Alzheimer’s	Attended regularly
C7	Atypical (56)			
C4	F (75)	Spouse	Mid-stage vascular	Attended regularly
C5	F (61)	Father	Diagnosis unclear	Volunteered regularly
		Mother	Late-stage Alzheimer’s	
C8	F (54)	Father	Mid-stage Alzheimer’s	None

As the interviewer, the first author closely monitored how respondents framed their answers, noting any differences of opinion. To avoid triggering arguments, follow-up questions were phrased carefully to sidestep direct contradictions. Caregivers talked more during the interviews, but their comments did not override those of people with dementia. The first author took care to empathize with both parties in cases of potential disputes and remained vigilant to intervene and redirect when caregivers unintentionally shifted toward their own viewpoints; these strategies are detailed in Dai and Moffatt (2021). Because of the difficulty of recruiting people with dementia, the varied manifestations of dementia (e.g. different types of dementia and individual symptoms), and the diverse situations of the participants with dementia and their caregivers, no data saturation was reached or pursued for the dyadic and caregiver interviews.

All interviews were conducted in library meeting rooms or participants’ homes, according to their preference. Each participant was compensated with $30 or a gift of approximately the same value, according to their preference. Each interview took 1–2 hours and was audio-recorded. Running over 17 hours in total, the recordings were fully transcribed with Amazon Transcribe, and then proofread manually.

### Data analysis

We conducted a thematic analysis (Braun and Clarke, 2006) on the observation notes and interview transcripts with NVivo 12. The first step involved inductive open coding, performed by the first author, guided by our two research questions; codes were based on the language participants used to describe their experiences. Then, the emerging codes were analyzed and synthesized into themes and subthemes. For example, a facilitator comment of Tales & Travels aiming “to provide them. . . with an environment where they’re safe,” was coded “safe environment” as an attribute of this program which later evolved with related codes into the subtheme “familiarity and safety in an inclusive environment” under the theme “a community platform.”

Taking the reflexive thematic analysis in a constructionist framing (Braun and Clarke, 2019), the authors went through an iterative process of revisiting the relevant data sections and regrouping the codes and excerpts, which allowed for finetuning of the themes and subthemes. After the data collection, the first author continued to attend Tales & Travels as a volunteer facilitator for ten sessions from September to December 2019 and speak at Alzheimer events. This prolonged immersion helped us continuously reflect on our interpretation of the context and our situating of the findings, deepening the reflexive approach to a thoughtful and repeated engagement with the data (Braun and Clarke, 2019).

Mindful that the voices of facilitators and caregivers could overshadow or deviate from those of people with dementia, we triangulated findings across observations and interviews, enabling interpretation of both alignments and contradictions in the data (Dai and Moffatt, 2021). For example, when a dyad’s positive Tales & Travels experiences were mainly recounted by the caregiver, we verified through observations that these accounts reflected the mutual satisfaction of the program.

## Results

The analyses led to two main themes. The first, “a community platform,” analyzes the attributes of this platform and how it supports engagement, fosters relationships, helps caregivers, and reaches out to broader communities. The second, “librarians’ roles,” explores the librarians’ close collaboration with professionals, comprehensive preparation and research, and evolving facilitation of the sessions.

### A community platform

Respondents discussed the library’s place in the community and their community-based experiences. Several commented on a range of community activities they sought, such as concerts, museums, or neighborhood walks, including the library and its programs. All dyads and caregivers noted the challenges in remaining socially active and the need to avoid isolation.

C5: So that changed a lot. [My parents] hardly left the house. . . The windows were never opened. . . they just existed within the boundaries of the walls of their home.

F4: . . . there is one woman who loves kids and really enjoys seeing children in the library, whether they’re just walking by the room. . . being out in public. . . is socializing, but they’re also taking a taxi and walking down the hall and seeing people. . . they’re engaging with the public as well as people in our program.

Many respondents emphasized how the library in general, and Tales & Travels in particular, are normal activities, and ones that participants had regularly engaged in.

F1: . . . some participants. . . quite often at liberty will just come and read books [or] magazines. Some of them are quite regular users of the library actually. . .. some participants don’t have that much going on. . .they’re happy to have that to come to.

Related to this was the absence of attention to or focus on dementia. Respondents highlighted how the emphasis on people coming together in a community setting to socialize contrasted with services oriented toward dementia, in more clinical settings, or under the specific guise of “Alzheimer’s” or “dementia.” F1 referred to Tales & Travels as “the program on the traveling discussions” instead of mentioning dementia directly to the participants. F2 regarded its uniqueness as “in a public place,” not “specially made for people with cognitive impairment.”

F2: . . . in the library, in a public place. It’s meant to make it look like. . . normal life. . . that’s when they actually enjoy about it. . .. they’re just going. . . somewhere like you would go to a reading club. . . it is labeled [dementia], but like it’s not out there and shown too much.

#### Familiarity and safety in an inclusive environment

Many participants were comfortable at a library venue and enjoyed the company of caregivers and facilitators in a cozy community atmosphere. As observed in video sessions, participants often moved freely in the room and chose where to sit while facilitators ensured that each participant had company. It resembled a big family enjoying afternoon tea and videos together. In this space, most participants freely engaged in the moment, for example, sharing when they had stories to tell and listening when they wanted to learn from others, as P1 described: “I really enjoyed travel. I want to find out more about these places. . . I tell stories if I’ve been to places. Otherwise, I’m listening to find out something.”

All facilitators commented on various attributes of this inclusive community platform, featuring familiarity, hospitality, safety, and adventurousness. The “unique,” “familiar feeling,” and “old memory” of a library reassured and encouraged people with dementia to attend “normalized” activities:

F4: One of the things that makes Tales & Travels unique is the familiarity of the library as the location to have the activity. It’s very normalized. . . even if someone doesn’t know where they’re going and you say, “Oh, we’re going to the library.” People know what the library is, and it’s an old memory. Usually, people have childhood memories of the library, so going to the library is a very familiar feeling.

Most facilitators mentioned the program attracting many returning participants, as C1 confirmed: “. . .whenever it’s on, we’re here. . . as much as we can.” The secure and affectionate feelings behind the choices of frequenting the program were highlighted:

P3: I want only [to] go with the people I’m secure with. . . They are wonderful. They always say “Hi.” Yeah, they’re very affectionate in many different ways.

C3: . . . You choose Tales & Travels.

A participant was observed saying: “Have you ever been there, this island? I want to go there. . .” and then later, “I’m old, but I still feel like a young man.” These comments demonstrated the forward thinking, young mindset, and hopefulness encouraged by the program’s familiar, safe, and inclusive environment.

However, challenges arose in offering activities for people with dementia in libraries or other public spaces. For example, F2 mentioned that the library, especially the bathroom, was not fully adapted and that more human resources were needed, for example, as a backup in case of an emergency.

#### Supporting engagement

Tales & Travels provided opportunities for participants to lead or join in a broader range of discussions and interactions inspired by the theme country. Facilitators were often observed not interrupting participants’ spontaneous, off-topic discussions. F3 pointed out that the best scenarios were participants “running the show” and engaging with materials and topics that interested them at the moment:

F3: [At] some of the best sessions. . . the clients are basically running the show, like they’re so interested in material or talking that it works. . . We were in in Madagascar. . . There’s a curiosity element in those few moments of our discussion that was really fun. . . they seem like, very interested in exploring. . . the animal side. . . wondering what they’re doing [or] thinking. . .

Most caregivers and facilitators stressed the social aspect of the program, engaging participants in interesting, and relaxing conversations:

C5: [Tales & Travels is] social. People like social. . . where they don’t feel alone. . . They’re sitting, and they’re talking. . . I don’t think it has to be an all-out intense activity. Just [something] that people can talk about.

The engagement extended beyond conversations to heartwarming occasions when participants engaged in singing, sometimes changing their mood from frustration to enjoyment:

F4: . . . we talked about France, and we played some old music by Edith Piaf, “La Vie en Rose. . .” she started to sing along. And her daughter. . . started to cry, because she. . .had never heard her mother sing, ever. . .that was really a special moment [that] changed her mood. . . from being frustrated. . . to singing and being calm and saying, “Thank you. I had a good time” when she left.

#### Fostering rapport

The program helped participants connect with peers, caregivers, and facilitators, with whom they sometimes formed lasting bonds. The first author observed regular participants greeting and chatting with each other, expressing how much they enjoyed their friendship verbally and non-verbally. A couple of participants often stayed afterward to chat more. Once, a participant held out her hands to another and said: “Just remember, I’m your friend.” Later, she added: “Friends are important.” Upon leaving, she hugged the facilitators and kissed them on the cheeks. All facilitators were open about their personal experiences when chatting with participants, building the rapport further:

F2: I don’t mind like sharing [my personal history]. . . they ask me where I’m from and everything, of course I will answer. It’s nothing so personal.

Such regular activities with familiar attendees helped ease the difficulties that people with dementia commonly experience with new faces and changing timetables. C1 points out this preference through discussion of past experiences dining on cruise ships:

C1: I think it’s harder for P1 if we have different people every night.

P1: No, it’s alright. Just as long as they want to chat. Some people do.

C1: I think that you do prefer it when you see the same people. . . The cruise line that we’ve been with most in the last few years [has] fixed hours and you have the same table for the whole time. So, that has become what we’ve become accustomed to.

#### Helping caregivers

Several caregivers reported benefiting from the program by expanding their support network, having some respite, or joining the activities. While remaining focused on participants with dementia, Tales & Travels interactively included participants and their caregivers. C3 appreciated that it was “great for everyone, the caregivers who attend also,” unlike other programs “sort of exclusive” to people with dementia. Alternatively, some caregivers chose to enjoy themselves, as they generally get few opportunities for time alone, which is “very very hard” to find (C6). For example, C4 would drop her husband off at Tales & Travels and browse the library: “I read my book. I enjoy it.”

Attractive and engaging, the program could calm participants down and make them feel at ease without their caregivers’ constant presence. Several participants were attracted to the activities and settling down from the initial anxiety of being separated from their caregivers. Once, a participant was anxious to find his wife at the beginning, repeatedly leaving his seat to look for her. However, once the table started talking about the materials, he gradually engaged in and enjoyed the conversations until the coffee break.

Admittedly, such a library program had limits in its aid to caregivers, as F2 mentioned that some participants needed more one-on-one attention and had to be accompanied by their caregivers. However, the first author sometimes witnessed caregivers chatting outside the Tales & Travels room, taking the opportunity to support each other. Together with other community activities, Tales & Travels enabled caregivers to connect with others:

C1: But we are making new friends through Tales & Travels. It’s very interesting. . . at the movie I saw somebody. . . that I knew from Tales & Travels. . . at the opening of Haida [exhibition], I saw someone else. . . I keep meeting people. Paths crossed.

#### Outreach

The library offered three suitcase kits with Tales & Travels materials to be loaned out, especially to local residences, for those who could not go to the library in person. F4 explained the motivation to develop these kits: “This program is missing some . . . people in this community living with dementia that cannot access our program.” Providing materials in suitcases was practical for delivering to home-bound community members.

F4: . . . a suitcase is a perfect way. . . they can literally leave the library and dragging a suitcase. . . with wheels. . . it is practical. . . home-bound residents in the community can borrow items, we select items for them, and then we deliver it to their home.

Such outreach was successful as the recreation facilitator at a local residence appreciated these kits and the librarians’ aid in providing new activities and materials:

F4: We tried to bring the Tales & Travels series out of the library to some local residences [to] people who cannot physically make it to the library and [are] more advanced in their dementia. . . I think the recreation facilitator over there really enjoys it, [and] they are always looking for something new. . . a lot of what they do is in-house animation [for which] this kit [provides] work that they would have had to do [and activities] they wouldn’t have even had the idea to do. . . she’s happy to say, “Okay, we’re going to do Greece. I’ll get it at the library.” [She] doesn’t have to print her own material.

A few caregivers expressed that such activities could potentially reach a broader community. C8 suggested “mobile Tales & Travels,” a specialized team bringing this “excellent activity” to different residences. C5 pointed out that it would be “amazing” to apply this effective “formula for success” of multisensory stimulants to senior residences:

C5: I was talking about Tales & Travels to my mother-in-law, [who is] completely autonomous. . . She said, “. . . I wish they brought this here.” I think that it’s a kind of activity that just would bring so much interest from the participants, who want to hear about different countries and be stimulated by. . . the facts. . . by the pictures, by the videos, by. . . a little taste of the country. I think it’s such a good formula for success in that population.

### Librarians’ roles

#### Collaborating with professionals

Tales & Travels was initiated in the library community and reinforced through collaborations with the Alzheimer Society. As F4 recounted, the library staff had “a heightened awareness” of the “aging community” and connections to dementia through patrons and their families. After the library director discovered the original program at a conference, the Alzheimer Society professionals guided them through the initial uncertainties in adapting the program.

Running Tales & Travels, the librarians closely worked with and constantly learnt from Alzheimer Society coordinators. They discussed table arrangements to better pair participants beforehand and recapped successes and challenges afterward. The librarians learned intervention strategies from experienced professionals, whose presence was “reassuring”:

F4: It was reassuring to have a professional who has experience with dementia, has strategies on specific interaction. . . I learned some of the strategy [that a coordinator] used to make people feel more comfortable, to defuse someone, maybe who is starting to get agitated or. . . stressed.

Notably, the librarians were not necessarily trained or experienced in dementia care, as F1 mentioned: “Lots of experience with the public, but not trained for this specific clientele.” Therefore, “a learning experience” was needed (F4). F1 reflected that several facilitators were needed for a busy session or in case of emergency, “at least one [facilitator] for five [participants].” Such a social program required facilitators to be trained in understanding participants, maintaining an interactive and stimulating structure, and actively participating in the conversations:

C5: . . . you need to have facilitators who understand the people sitting at the table and how to get them involved. . . if you don’t have that kind of structure. . . it could lack stimulation for the participants. . . The facilitators [need] training as to how to handle the table, how to get people involved. . . you need to actively engage them. If you don’t, [they will] get lost in their own thinking and not get anything out of it. . . the way it’s run in the [library has] active participation from the facilitators at the tables.

On the other hand, working with librarians rather than other dementia professionals provided Alzheimer Society coordinators with opportunities for “a different approach” and enriched the activities in an “intellectually stimulating” way that F2 appreciated as a learning opportunity for everyone.

As the program matured F4 sometimes trained recreation professionals at local residences about how to use program materials so that “they got a sense of how it works” and would be able to “take the kit” and “run the program by themselves.” Expanded collaborations between librarians and various professionals can potentially reach broader communities, where awareness and training regarding dementia care were needed:

C8: [It] would be better for. . . activities director knew more about the disease. . . [If] staff in residences. . . understood the disease better, they would probably create activities that were. . . better suited for people with dementia.

#### Preparing and researching

Running Tales & Travels, the librarians were in charge of organization, registration, preparation, and research before each session. They prioritized promising conversational starters (e.g. questions and “fun facts”) and gathered suitable books and large print images. They ensured a balanced distribution of these materials across the tables, with the same sets of images but different books.

F4: . . .children’s fact books about the country [are] very easy to find facts and more images, which actually bring a good connection between the information and the book. A good way to absorb the information is with images. . . adult books will [be] coffee-table style.

During coffee break, snacks from the featured country were a routine, and traditional instrumental music of the featured countries was sometimes brought in “to create an ambiance” (F1). Travel guide videos were also carefully chosen in terms of length and style:

F1: We usually try to pick short videos from four to six minutes top. . . comprehensive [clips like] National Geographic. . . are very good at giving information about these countries, and others could be. . . landscapes. . . with music.

The preparation process mobilized a range of librarian skills to meet participants’ needs. F1 reflected that preparing for Tales & Travels was “fun” but “stressful” at first because it was a unique program for an unfamiliar audience and required suitable ways to present materials and structure information:

F1: . . . at first, I think I was a bit tested in the way that I would present things. Don’t put too [many] words. Don’t overwhelm them with information. Go to the essential, the more. . . catchy stuff.

Care was taken to avoid negative topics and materials, such as wars, chaos, social economic problems, presenting “more in a touristy way” (F1). Each librarian had their own preparation approaches, and the program “grew organically” (F4). As librarians put efforts in preparation and “felt that it was something good that we were offering” (F1), most caregivers applauded their “good job” of running the program, conducting “wonderful research,” and developing new components such as fun facts about countries, “an excellent thing to have” (C1).

All facilitators explored multisensory materials and had an awareness of different manifestation and advancement of dementia. F2 suggested “more sensory activities” for participants in later stages to promote touching and listening to music. Themed snacks were particularly appreciated:

F3: The more themed the better. . . having a savory element, sometimes you get a little closer to a country’s food flavors. . . Food is an important component to animating longer sessions, period. It’s such a nice break. . . such a normal thing to do with people. . . It’s a perfect time to socialize. . . you’re relaxing, but at the same time you’re discussing. . . you greet people with coffee at the beginning or water. . . They can boost up the energy a little bit.

#### Facilitating

At Tales & Travels, the librarians’ work extended beyond their usual organizers’ role to facilitating each session in person, requiring “a lot of effort weekly” but “very rewarding”:

F4: [Tales & Travels is] very, very rewarding. . . it’s a lot of effort weekly. . . [If you run] a lecture for the public, you have to do your research, find out who to invite, [coordinating]. But once it happens, the lecturer speaking and [your job is done]. . . [Somewhat similar to] the way some children’s librarians [do] story times themselves, but then they have other people coming run activities. There’s that balance. . . you feel the positive feeling. . . the results quickly in a way.

F4 further detailed how they arrived at the weekly sessions to create a “regularity” for participants, attended to details of each component along the way, adjusted the table arrangements according to attendees of each session, and recognized the importance of “being flexible and adaptable” overall. Similarly, F1 reflected on the evolving nature of their facilitation, retaining effective methods and exploring solutions to emerging problems: “It evolves. . . If it works, we’ll keep it. If it doesn’t work, we try new things and see what is best usually.”

The librarians made a point of getting to know the participants, explored personalized ways of communication, and added a personal touch in choosing the materials, topics, and activities. This person-centered approach attended to individual differences, welcomed storytelling alongside listening, and made everyone “comfortable in the environment”:

F1: When you start knowing your participants, that’s really when you know how to interact with each of them. . . it’s really individually different. It depends on the person, how they want to share their information, their stories, or if they don’t want to share them but they’re happy listening to others. . . I feel like that’s how to create a good ambiance, and everyone feels comfortable. That’s the main goal, and everybody is happy to be there, and they’re comfortable in the environment.

C5 confirmed the personalized approaches to engaging participants and keeping everyone in the conversation:

C5: Sometimes you feel that you’re losing some of them. So, when you know them. . . if [one participant] not participating, I’ll show her a picture of an animal and it just kind of brings her back. Or you ask someone else to read because. . . that gets them involved. . . Or you ask another person [if] they’ve traveled there. So, it’s a way of making sure that. . . they all participate in a way, and they all feel like you’ve given them that little extra attention. . .

Many caregivers applauded the librarians’ efforts. C7 pointed out two factors attracting them to Tales & Travels, the venue (“[the Library], an establishment that I respect”) and the librarian who led the program. P1 and C1 especially praised F4’s essential role in making the program unique and engaging:

C1: I think partly it’s F4 running it. . . he has such an amazing way of presenting things and welcoming people that. . . his personality really helps to bring the groups together.

P1: It’s nice to talk to him too when he opens up.

The facilitation roles brought various challenges. If one talkative participant was “monopolizing” the conversation, facilitators needed to manage the situation and find ways to “give space for people” to contribute (F3). Facilitators carefully dealt with “subtle” situations, mediating emerging disputes and managing participant dynamics:

F1: . . . it was subtle. . . aggressive things were said and then the person was just overwhelmed and decided to leave. . . [Someone] laughs very loudly and. . . irritated someone else, so they didn’t want to sit together. [You] see the dynamics once you start to know the patrons. . .

## Discussion

The findings demonstrate how library services expand community platforms for dementia care and diversify librarians’ roles. This section first draws from the results to answer the two research questions, while the third subsection reflects on the study limitations and opportunities for future work.

### Enriching community-based dementia care

#### Furthering inclusivity and engagement in community programs

The findings reveal that public libraries are well-positioned to reach people with dementia and their families to promote inclusive and engaging social activities. Tales & Travels offers a community platform for participants to spend time in a public space and enrich their daily lives through library resources and interactions with others. Through mature and intellectual activities in a normalized setting (Dai and Moffatt, 2020), Tales & Travels welcomes people with dementia and their caregivers as community members. It illustrates opportunities for community programs to help both care recipients and caregivers adjust daily routines and keep socially active. These findings echo the concept of “carer-friendly” highlighted in recent work on museum programs for people with dementia, suggesting careful considerations of caregivers’ needs and impact on the process and facilitation of activities (Kinsey, 2021).

Whereas library services for older populations have been well investigated (e.g. Bennett-Kapusniak, 2013; Cavanagh and Robbins, 2012), more attention and specific guidance are needed for developing dementia-related programs. Tales & Travels reveals promising avenues to balance person-centered attention and group engagement, such as peer collaboration and open-ended experiences (Dai and Moffatt, 2020). Likewise, fiction-reading activities could facilitate creativity and enjoyment (Rimkeit and Claridge, 2017). Positive effects of literature-based intervention for dementia (Billington et al., 2013) can be extended from clinical settings to communities, offering sustainable, long-term engagement.

These inclusivity and engagement efforts will help reduce the stigma and negative social impact attached to dementia (Riedner et al., 2020), further demonstrating the role of public libraries in community building through encouraging social inclusion and equity (Scott, 2011). Library services can contribute to expanding the contemporary concept of dementia-friendly communities beyond healthcare settings (Lin, 2017). Future research and programs could be enriched through diversified framings, such as creative stories (Vanderpool and Vanderpool, 2017) and the notion of play in innovative arts-based approaches to dementia care (Swinnen and de Medeiros, 2018).

#### Extending accessible and collaborative library services

This case study, along with previous works, highlights accessible library services for diverse populations including people with disabilities and complex needs. As exemplified by the Tales & Travels suitcase kits, the delivery of well-selected materials to local residences and home-bound community members can provide easier access to a broader audience.

This outreach echoes health sciences libraries’ efforts to reach out to family caregivers (Howrey, 2018) and could further enrich library services such as the “lost voices” project helping people with memory loss record their own oral histories (Farthing and Davies, 2017). Public libraries’ community outreach expands collaborative social services, through notable works on social and information networks of homeless populations (e.g. Hersberger, 2003). More recently, public libraries’ responses to the opioid crisis extend their collaborative services through partnerships with public health services, professional associations, and nonprofit organizations, leading to effective strategies for forging partnerships and meeting community needs (Coleman et al., 2020).

Within broader conversations on public librarianship, this case study demonstrates the appreciation of the library as a provider of community-based programs by expanding collaborative services and partnerships. As shown at Tales & Travels, public libraries offer community space accessible to all population groups, instead of explicitly associated with healthcare or clinical settings. Approaching libraries as sites of community activities further opens space for librarianship research directions such as community-led library services (Pateman and Williment, 2013), asset-based community development approach (Stevenson, 2020), and integrating communities of practice into library services (Kim, 2015).

### Leveraging public libraries’ strengths and navigating challenges

#### Awareness, collaboration, and resources

Tales & Travels results from the awareness of community needs, as a growing number of library patrons have dementia-related experiences. Libraries are becoming partners in government efforts to build dementia-friendly communities, making a difference to people with dementia (Alzheimer Society of British Columbia, 2016). As respected community institutions, libraries are vital to a dementia-friendly community by providing access to care resources, services, and programs (Dementia Friendly America, 2016). To raise broader awareness, libraries could hold themed activities or months for mental or physical conditions, with careful selection and justification of the themes (Kowalsky and Woodruff, 2017).

Building upon the original program and the collaboration with local Alzheimer Society professionals, Tales & Travels has matured as a well-received series and expanded its community impact. Library services are poised to respond to communities’ changing needs in collaboration with a variety of professionals, achieving mutual benefits, as this study shows that Alzheimer Society coordinators appreciated working with librarians. These collaborations expand library dementia services, from stimulating memories (e.g. through reminiscence kits) to offering enjoyment and entertainment (Mortensen, 2007).

The case study underlines the library resources that could be mobilized for developing and running such programs, starting with a range of materials usually abundant at libraries (e.g. books, printed materials, music, videos, and open spaces). As found in other community outreach programs, librarians play an integral part and are of great value to the partnerships (Basler, 2005). Even though they might not have the opportunity to receive official training for specific dementia care needs, librarians’ skillsets and professionalism help them successfully navigate the learning curve and facilitation challenges when serving the dementia community. To aid in program planning and staff training, more concrete guidelines on interacting with people with dementia could benefit library services (Baker et al., 2018). Recent works on designing environments for dementia (Bowes and Dawson, 2019) and programming for dementia and caregivers (Dickey, 2020) are informative resources.

#### Further considerations for practice

This case study reveals some best practices as starting points for enhancing library services in this space. Tales & Travels participants confirmed a feeling of security as a key factor, along with the staff’s warm hospitality, in choosing venues and activities. Building upon safety and inclusivity, the program further offered opportunities to envision future tourist adventures and remain mentally youthful, an element less discussed in dementia care. Looking back and remembering the past provided one way of engaging participants, but the future could be in the picture as well.

Tales & Travels benefits from deemphasizing dementia and exploring sensory activities that speak to the audience at different stages of dementia. Providing refreshments, preferably themed, has proven effective. Savory elements can create socializing opportunities and keep the participants refreshed, adding to the cozy feelings of normalcy. Other effective strategies adopted by the facilitators include giving attention to participants in a respectful, person-centered way, balancing the subtle dynamics among patrons, and making space for everyone to participate and contribute. Meanwhile, more attention is needed to the physical settings to ensure facilities are adapted for accessibility.

### Limitations and future work

As this study is set in a populous municipality in Canada, some findings might have inherent social and demographic limitations when applied to other communities. Although the respondents come from various cultural, educational, and professional backgrounds, most of them have higher education and comfortable socioeconomic status, which may have pre-conditioned their active involvement in social events and research. Future work can diversify perspectives across various communities and programs.

Given the physical distancing restrictions in the COVID-19 pandemic at the time of this writing, the Westmount Public Library started offering an online version of Tales & Travels in February 2021. This new adaptation opens opportunities to investigate library programming for dementia care in virtual settings. Our previous work has noted that events physically held at libraries might hinder participants with limited mobility, whereas technology-mediated activities could conversely reach a broader audience yet lose the benefits of public, tangible environments (Dai and Moffatt, 2020). Future work can compare the two versions of Tales & Travels and delve deeper into tensions between physical and virtual spaces.

## Conclusions

The fact that libraries can contribute to healthcare through programs created for specific populations might not be a common association, and yet, such programs have shown great results and potential. This paper presents a case study of Tales & Travels, a storytelling and social program for people with dementia run by a public library in Canada. Interviews and observations integrate and examine viewpoints of this program and broader social activities from various stakeholders including librarians, Alzheimer Society professionals, people with dementia, and family caregivers.

By analyzing library contributions to community-based dementia care (RQ1), this study identifies inclusivity, engagement, accessibility, and collaboration as key factors. It demonstrates that library programs and outreach can enrich activities for people with dementia and their caregivers, extending partnerships between libraries and other agencies. By assessing libraries’ strengths and challenges in running such programs (RQ2), the study highlights librarians’ awareness of the community’s evolving needs and their collaboration with other professionals. It provides examples of useful resources and practical considerations for mitigating emerging challenges. In sum, this paper offers insights immediately applicable in practice and advances librarianship research on library services for dementia care. Hopefully it will inspire other initiatives in which information professionals can help improve the well-being of vulnerable populations.

## Supplemental Material

sj-docx-1-lis-10.1177_09610006211065170 – Supplemental material for Library services enriching community engagement for dementia care: The Tales & Travels Program at a Canadian Public Library as a case study Click here for additional data file. Supplemental material, sj-docx-1-lis-10.1177_09610006211065170 for Library services enriching community engagement for dementia care: The Tales & Travels Program at a Canadian Public Library as a case study by Jiamin Dai, Joan C. Bartlett and Karyn Moffatt in Journal of Librarianship and Information Science

## Notes

1. To protect the anonymity of the facilitators, we reported their backgrounds collectively, instead of detailing on the individual level.

## Anonymized materials

1. Introduction, the last paragraph, the original first sentence: “This case study is situated in an adaption of the original Tales and Travel Memories series run by the Westmount Public Library in greater Montreal, Canada, in collaboration with the Alzheimer Society of Montreal.”
2.1 Dementia-Friendly Library Services, the last paragraph, the original last sentence: “Our earlier work, which focused on identifying technological opportunities for promoting community-based social sharing. . . (Dai and Moffatt, 2020).”
5.3 Limitations and Future Work, the original last paragraph: “Given the physical distancing restrictions in the COVID-19 pandemic at the time of this writing, the Westmount Public Library started offering an online version of Tales & Travels in February 2021. This new adaptation opens opportunities to investigate library programing for dementia care in virtual settings. Our previous work has noted that events physically held at libraries might hinder participants with limited mobility, whereas technology-mediated activities could conversely reach a broader audience yet lose the benefits of public, tangible environments (Dai and Moffatt, 2020). Future work can compare the two versions of Tales & Travels and delve deeper into tensions between physical and virtual spaces.”
