# Supplementary material for: Library services enriching community engagement for dementia care: The Tales & Travels Program at a Canadian Public Library as a case study
Source: J Librariansh Inf Sci. 2021 Dec 29;55(1):123–36. doi: 10.1177/09610006211065170 (PMC10015598; doi:10.1177/09610006211065170)
Supplement: sj-docx-1-lis-10.1177_09610006211065170 – Supplemental material for Library services enriching community engagement for dementia care: The Tales & Travels Program at a Canadian Public Library as a case study [file sj-docx-1-lis-10.1177_09610006211065170.docx]

# Library Services Enriching Community Engagement for Dementia Care: The Tales & Travels Program at a Canadian Public Library as a Case Study

# Appendix – Facilitator, Dyadic, and Caregiver Interview Guides

# Facilitator Interview Guide

[Self-introduction] We are investigating how to better support people with dementia sharing stories and socializing, especially at events such as Tales & Travels. There are no right or wrong answers. You can skip any questions that you don’t want to answer. You can stop or take a break whenever you want. Participation is confidential, and your name will be replaced by a number. Thank you for helping us and sharing your experience in facilitating the Tales & Travels sessions.

1. To start, how long have you worked with individuals with dementia?

*For coordinators:* What kind of training have you had about dementia?

*For librarians:* Have you had any training about dementia? What kind?

1. When did you start to facilitate the Tales & Travels program?

How many sessions have you facilitated?

1. Could you walk me through your preparation process for the last session of Tales & Travels that you facilitated?

Why did you choose this type of material (images/facts/books/food/music/video)?

1. Could you walk me through the last session of Tales & Travels that you facilitated?

How many people sat at your table during the story session?

Why did you choose to arrange it this way?

1. What changes did you make to the materials or procedures along the way?

Why did you make this change?

1. How did you encourage participants to tell their stories?

How did they respond to these prompts?

1. Could you recall the feedback/comments you got from the participants after a session?

Any comments from their caregivers?

1. Could you tell me about a time when something went really well during the session?

Do you remember any interesting discussions among the participants or with you?

1. Could you think of any difficulties you had during the sessions?

Anything didn’t go well as expected?

1. What challenges have you observed when participants tell stories?
2. What challenges have you observed when participants socialize with each other?
3. Did you notice any difference in the conversation when the caregiver sat at the same table?
4. We've talked about telling stories.

Did you recall if there was anything else that the participants like to share with others?

Books, music, or movies they like? News and sports? Food?

1. Did you notice any other activities that participants engaged when they came to the library, before or after the Tales & Travels sessions?
2. Do you recall dementia being mentioned during the sessions? What was the occasion?
3. Looking back now, do you have any thoughts about what would make a successful session?
4. What positive outcomes did you observe from your facilitating experience?
5. Have you facilitated or participated in other activities for people with dementia?

Anything unique you only observed in the Tales & Travels?

1. Let’s say you have unlimited resources, what activities would you add to the current sessions?

What equipment or support would you like to have?

1. Is there anything you would like to add? Have we missed anything you think is important?

# Dyadic Interview Guide

[Self-introduction] We would love to hear stories and thoughts from both of you about your social experiences. There are no right or wrong answers. You can skip any questions that you don’t want to answer. You can stop or take a break whenever you want. Participation is confidential, and your name will be replaced by a number. Thank you for helping us and sharing your stories.

1. To start, I’d like to know a little bit about you.

How did you two meet / know each other?

How did you like to spend time together back then?

May I ask how long have you been retired?

How did you like to spend time together when you just retired?

How about now?

1. I’d like to know a little bit about your daily life. Could you walk me through your last week? We could start with Monday July 15.

What was a typical week of yours like? From Monday to Sunday.

1. Do you often go to social occasions now?

- For example, family gatherings?
- Meeting with friends? How does that compare to family gatherings?
- Public events? Something related to your hobbies? Entertainments?

1. How about a few years ago / when you just retired?

Did anything change in your social lives over the recent years?

1. Could you tell me about a time when you really enjoyed a social event?

Which part of the event did you like the best?

1. What do you like to discuss with other people at social events?

Do you prefer to have a conversation with one person or several people together? One-on-one or group discussion?

1. Could you tell me about a time when a social event was unpleasant or awkward? Something that made you uncomfortable or made a conversation difficult? It could be the little things, or just an unpleasant moment.

What bothered you? How did you deal with it?

1. The Library organizes the Tales & Travels event, exploring a country every week, with the help of images, maps, books, food, and videos. How long have you been attending this event?

What do you like the most? What’s the best part for you?

In your opinion, what makes T&T unique / different from other events?

1. Have you attended any similar event? Could you tell me about it?

Could you give me a concrete instance of talking about yourself / sharing your stories with others? For example, some people carry pictures of their grandchildren and share with others.

1. We’ve talked about sharing stories.

Is there anything else that you like to share with other people?

Your hobbies, skills, expertise? Books, music, movies you like? News, sports, or food?

1. Are there any activities you’d like to add to your current daily routine?

Attending some events more regularly? Visiting some people/places more often?

1. Let’s say you are organizing a party and you could have all the money and help you need. Unlimited resources. Whatever you want. What would you do to make it successful? What activities would you like to plan? What equipment or service would you like to have? What would the ideal space look like? How many people would you like to invite?
2. Is there anything you would like to add about your social lives?

Have we missed anything you think is important?

# Caregiver Interview Guide

[Self-introduction] We are trying to learn about the social experiences of people with dementia and their caregivers. We would love to hear your stories and thoughts. There are no right or wrong answers. You can skip any questions that you don’t want to answer. You can stop or take a break whenever you want. Participation is confidential, and your name will be replaced by a number. Thank you for helping us and sharing your stories.

1. To start, I’d like to know a little bit about you and your caregiving experience.

How long have you been a caregiver? Who are you taking care of?

How did you like to spend time together a few years ago?

What did you like to talk about with each other then?

How about now? Has this always been the case?

1. I’d like to know a little bit about you and [the care recipient]’s daily life. Could you walk me through your last week? We could start with last Monday.

What was a typical week of yours like? From Monday to Sunday.

1. Did you and [the care recipient] often go to social occasions in recent years?

Who do you meet more often? Who are you in frequent contact with?

- For example, family gatherings?
- Meeting with friends? How does that compare to family gatherings?
- Public events? Something related to your hobbies? Entertainments?

1. How about a few years ago? Did both of you go to a lot of socials back then?

Did anything change in your social lives over the recent years?

1. Could you tell me about a time when both of you really enjoyed a social event?

Which part of the event did you like the best?

1. What does [the care recipient] like to discuss with other people at social events?

Does he/she prefer to have a conversation with one person or several people together?

1. Could you tell me about a time when a social event was unpleasant or awkward? Something that made [the care recipient] uncomfortable or made a conversation difficult? It could be the little things, or just an unpleasant moment.

What bothered him/her? How did you deal with it?

1. The Library organizes the Tales & Travels event, exploring a country every week, with the help of images, maps, fun facts, books, food, music, or videos. Have you attended this event?

What do you like the most? What’s the best part for you?

In your opinion, what makes T&T unique / different from other events?

1. Have you attended any similar event? Could you tell me about it?

Could you give me a concrete instance of talking about him/herself, sharing his/her stories with others? For example, some people carry pictures of their grandchildren and share with other people.

1. We’ve talked about sharing stories.

Is there anything else that [the care recipient] likes to share with other people?

Hobbies, skills, expertise? Books, music, movies? News, sports, food?

1. Are there any activities you’d like to add to [the care recipient]’s current daily routine?

Attending some events more regularly? Visiting some people/places more often?

1. Let’s say you are organizing a party and you could have all the money and help you need. Unlimited resources. Whatever you want. What would you do to make it successful? What activities would you like to plan? What equipment or service would you like to have? What would the ideal space look like? How many people would you like to invite?
2. Is there anything you would like to add about your social lives?

Have we missed anything you think is important?
